# Using online search activity for earlier detection of gynaecological malignancy

**DOI:** 10.1186/s12889-024-17673-0

**Published:** 2024-03-11

**Authors:** Jennifer F. Barcroft, Elad Yom-Tov, Vasileios Lampos, Laura Burney Ellis, David Guzman, Víctor Ponce-López, Tom Bourne, Ingemar J. Cox, Srdjan Saso

**Affiliations:** 1 Imperial College London, Hammersmith Hospital Campus, Du Cane Road, London, W12 0HS UK; 2 Microsoft Research, Hoshaya, Israel; 3 Department of Computer Science, University College London, London, UK; 4 Computer Science, University of Copenhagen, Copenhagen, Denmark

**Keywords:** Ovarian neoplasms, Endometrial neoplasms, Early detection of cancer, Cancer screening test, Internet, Health

## Abstract

**Background:**

Ovarian cancer is the most lethal and endometrial cancer the most common gynaecological cancer in the UK, yet neither have a screening program in place to facilitate early disease detection. The aim is to evaluate whether online search data can be used to differentiate between individuals with malignant and benign gynaecological diagnoses.

**Methods:**

This is a prospective cohort study evaluating online search data in symptomatic individuals (Google user) referred from primary care (GP) with a suspected cancer to a London Hospital (UK) between December 2020 and June 2022. Informed written consent was obtained and online search data was extracted via Google takeout and anonymised. A health filter was applied to extract health-related terms for 24 months prior to GP referral. A predictive model (outcome: malignancy) was developed using (1) search queries (terms model) and (2) categorised search queries (categories model). Area under the ROC curve (AUC) was used to evaluate model performance. 844 women were approached, 652 were eligible to participate and 392 were recruited. Of those recruited, 108 did not complete enrollment, 12 withdrew and 37 were excluded as they did not track Google searches or had an empty search history, leaving a cohort of 235.

**Results:**

The cohort had a median age of 53 years old (range 20–81) and a malignancy rate of 26.0%. There was a difference in online search data between those with a benign and malignant diagnosis, noted as early as 360 days in advance of GP referral, when search queries were used directly, but only 60 days in advance, when queries were divided into health categories. A model using online search data from patients (*n* = 153) who performed health-related search and corrected for sample size, achieved its highest sample-corrected AUC of 0.82, 60 days prior to GP referral.

**Conclusions:**

Online search data appears to be different between individuals with malignant and benign gynaecological conditions, with a signal observed in advance of GP referral date. Online search data needs to be evaluated in a larger dataset to determine its value as an early disease detection tool and whether its use leads to improved clinical outcomes.

**Supplementary Information:**

The online version contains supplementary material available at 10.1186/s12889-024-17673-0.

## Introduction

Ovarian Cancer remains the most lethal gynaecological malignancy, largely because most women (75%) present with advanced stage disease [1]. Detection of ovarian cancer at an early stage is associated with a seven-fold increase in five-year survival (93 vs 13%), compared to advanced disease [1]. Endometrial cancer is the commonest gynaecological malignancy in the UK with a rapidly increasing incidence driven by the obesity epidemic [2, 3]. Despite advances in diagnostics only 38% of ovarian and 79% of endometrial cancers are detected at an early stage (stage I & II) [4]. Earlier cancer detection relies upon prompt referral for investigation of symptomatic individuals and periodic screening of the asymptomatic population.

The non-specific, vague symptoms associated with ovarian cancer, coupled with a poor awareness of its presentation among the general population, contributes to delayed diagnoses [5]. Primary care physicians (GPs) are responsible for identifying high risk patients and arranging urgent referral into specialist services for further investigation via an “urgent cancer pathway” [6, 7]. Referral barriers within primary care are known to be a key contributor to delayed cancer diagnoses, as women typically present to their primary care provider three times before being referred [6–8]. Reluctance among primary care clinicians to refer patients for further investigation is closely correlated to poor cancer survival rates [6].

An effective screening strategy can address primary care referral barriers, but requires engagement in the value of screening, which can vary significantly [9]. Whilst screening has proved effective in facilitating the earlier detection of cervical cancer and preventing 70% deaths in England, a cost-effective screening program for ovarian and endometrial cancer does not exist [10]. Two large randomised controlled trials (UKTOCs and PLCO), assessed the value of combined imaging (pelvic ultrasound) and a tumour marker (CA-125) to improve the earlier detection of ovarian cancer [11, 12]. Neither trial resulted in a mortality benefit, therefore were not deemed to be a cost-effective screening strategy by the National Institute for Clinical Excellence (NICE, 2011) and were not integrated into clinical practice. More recently, the Refining Ovarian Cancer Test Accuracy Scores (ROCkeTS) trial, which incorporates symptom questionnaires, serology, with ultrasound-based diagnostic models, is being evaluated in a prospective study, to determine their role in the diagnosis of ovarian cancer [13]. The value of routine pelvic ultrasound in endometrial cancer screening was evaluated in a systematic review of 11,000 asymptomatic women. It concluded that assessment of endometrial thickness has no value in endometrial cancer detection because of the inherent diagnostic inaccuracy in its measurement and was therefore not recommended [14, 15].

Widespread access to the Internet globally (97.8% in the UK) has facilitated vast numbers of online services [16, 17]. Users of these online services generate digital footprints either directly, through posting on social media platforms, or indirectly, through online search histories stored by service providers, such as Google. Google search has 86.3% of the UK online search engine market [18]. Worldwide, Google processes approximately 9 billion searches per day, of which 630 million are health-related (7%) [19, 20]. Digital footprints have been shown to be useful in disease surveillance [21, 22], and for generating individualised health risk profiles [23]. The non-episodic, temporally dense nature of digital footprints complements the conventional method of disease detection using sparse, episodic healthcare records. Online search data has identified individuals at risk of developing common health conditions, including myocardial infarction, allergies and Human Immunodeficiency virus, and in highlighting novel disease risk factors, useful in the prevention of disease [23]. The use of online search data to facilitate the earlier detection of cancer was demonstrated by *White *et al. [24], which showed that 58% of individuals, thought to have lung cancer (based on their online searches), could be identified using a predictive model (AUC of 0.89) up to 39 weeks prior to being diagnosed [24]. A similar finding was reported in individuals thought to have pancreatic cancer (based on their online searches) *by Paparrizos *et al*.* [25] Whilst the presence of a signal in online search patterns to enable the earlier identification of disease is exciting, previous studies used a ‘proxy’ diagnosis of cancer, based on an individual’s online searches, not a clinically confirmed diagnosis. This assumption may be invalid, as no study has clinically validated experiential searches in individuals with confirmed diagnoses. Furthermore, the interpretation of the disease timeline is limited by a lack of robust disease diagnosis time points.

Existing research in this field has focused on comparing a malignant cohort to ‘healthy population’ controls, rather than using a cohort with an underlying benign diagnosis. This has not only limited the comparison between benign and malignant conditions but is also likely to have resulted in overly optimistic findings.

We aimed to appraise online search patterns in symptomatic individuals with known gynaecological diagnoses, to determine (1) if there is a difference in online search patterns between individuals with a malignant and benign diagnosis and (2) if this can enable the identification of individuals with a gynaecological malignancy at an earlier stage.

## Methods

### Recruitment and inclusion criteria

This pilot study was conducted at a tertiary London University Hospital between December 2020 and July 2022. Women (aged 18 or older) who were referred to the hospital by their primary care physician (GP) with gynaecological symptoms, had a Google account and were English speakers were eligible to participate. Patients with a personal history of ovarian and endometrial cancer (previously treated) were also eligible for inclusion.

The requirement of a Google email account was necessary for this study, as the participant’s online search history was obtained through Google Takeout. Online search histories could be obtained using alternative search engines however Google was used for this study as it is the most common search engine in the UK. Informed written consent was obtained by J.B. or L.B.E to complete enrolment.

Within the National Health Service (NHS) women who attend their General Practitioner (primary care provider) and are felt to be high risk of a suspected cancer (based on symptom presentation) are referred to hospital (secondary care) for an urgent appointment within two weeks. Patients referred via this ‘urgent cancer pathway’ were eligible for recruitment into this study. Patients that consented to study participation completed a clinical questionnaire and extracted their Google takeout file.

Clinical outcomes (benign or malignant) for the study participants were extracted from the medical records, either clinical (ultrasound-based) or histological diagnosis, for those who underwent surgery. We analysed 24 months of Google takeout data prior to the GP referral date (see also below) and correlated it with the clinical outcomes (benign or malignant).

### Patient and public involvement

We involved patients in the study design, which informed changes to the questionnaire and terminology used in patient information sheets. It was a useful process to gain insight into patient opinion on using patient data to facilitate the earlier detection of disease.

### Online search data acquisition

Online search queries were extracted as a Google takeout file, which was shared with the research team via secure email. The Google takeout file was pseudo-anonymised. An automated health filter was applied to extract specific health-related queries for a 24-month period prior to the date of GP referral. The automated filter used a previously developed list of medical terms, comprising symptoms, disease, and drugs [23]. Only search queries which contained one or more of the keywords defined in the medical terms list remained in the filtered Google takeout file. Note that since this filter considered each word in a medical phrase, such as “club foot”, independently, the filtered output contained many, possibly irrelevant, queries, e.g., queries for “club”. The manual filtering process excluded queries pertaining to pets (e.g., “cat bleeding pain”) and to irrelevant medical conditions and body organs (e.g., “finger bleeding pain”). Queries that were repeated verbatim in consecutive searches were also eliminated.

Recognising the impact of the GP consultation on online search patterns, we evaluated online search data up to the day before GP referral i.e., excluded the date of GP referral from the analysis. Patients who were not referred by their GP i.e., presented to the emergency department or another specialty were included, but the date of consultation was used as a substitute for the GP referral date.

**Fig. 1:**
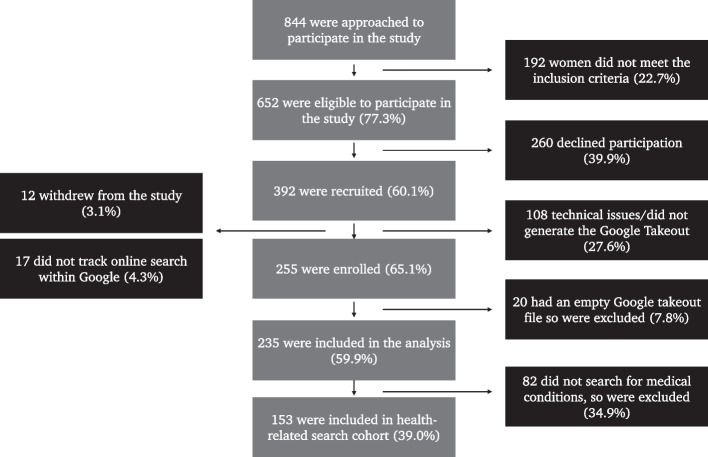
Summary of patient enrolment flowchart. The flowchart outlines the enrolment process for the study cohort (*n* = 235) and health-related search cohort (*n* = 153), from individuals referred to a London University Teaching Hospital with a suspected cancer between December 2020-June 2022. It outlines the reasons for incomplete enrolment and exclusion from the study

The key outputs included: (1) the time of first search query (2) the number of queries before filtering, and (3) the number of queries after the health filter was applied. Patients with Google takeout files (post filtering) that were empty were excluded from the analysis (Fig. 1).

### Clinical questionnaire data

A clinical questionnaire (Supplementary Fig. 1, Supplementary Table 1) was completed by patients to extract relevant clinical data, including demographics (age, BMI, ethnicity), medical and family history, and the nature of their symptoms at presentation to the clinic. Patients were asked if they had experienced up to 20 common gynaecological symptoms, their frequency, severity, and duration in the 12 months prior to presentation to the Gynaecology clinic. Relevant information was extracted from the medical records, including clinical (ultrasound-based) or histological diagnosis, for those who underwent surgery. Further details about staging and grade were available for malignant cases.

### Preliminary analysis of keyword categories

A list of health-related keywords was defined using known medical and colloquial terms, in English, French and Spanish, to allow inclusion of Google takeout file’s which were in French, Spanish, or English. The keywords were categorised manually by J.F.B and S.S into 14 categories (not mutually exclusive) (bleeding, bloating, diagnostics, fatigue, gastrointestinal, gynaecological, menopause, nutrition, other conditions, pain, pelvic organs, symptoms, urinary, vagina or pelvic organs) and non-relevant keywords were removed (Supplementary Table 2). Preliminary analysis of the online search data (search terms and clinical data) was performed by grouping search queries into categories (categories model) or individual words/word pairs (that have been used at least five times) and analysed their appearance over time. The resulting lists of query categories, keywords, and most common queries for each condition are presented in Supplementary Table 2*.*

### Prediction of outcomes

Patient outcomes (malignant or benign) were predicted using query data between T_1_ and T_2_ days prior to the GP referral date (T_1_ < T_2_), for patients that made online search queries during that time window. We considered all pairs of T_1_ and T_2_ between -700 days and -1 days to create different time windows. The input representation to the model was either based on categories or search terms:

1. Categories model: The number of search terms in each of the keyword categories, as described above.
2. Terms model: A vector-space model [26] of all words and word pairs (consecutive words) which were used by at least five patients. This representation is commonly used for text, especially when the terms with high information pertaining to differentiating between classes is unknown.

The categories model is considered to be based on expert knowledge, whereas the terms model is a data-driven approach including any terms used by more than five individuals within our cohort.

Predictor performance was superior in the vector-space model [26] and we therefore focus on this representation. Patient representations were used as input to a gradient boosting model, considered one of the state-of-the-art models for structured data such as the vector-space representation [27, 28], with 50 weak learners. The models were evaluated using leave-one-out cross-validation [29] to reduce the likelihood of overfitting. Leave-one-out is appropriate due to the size of the dataset [30]. In line with previous work [25, 31–34], the performance measure reported is the Area Under the receiver operating Curve (AUC). The output of the model is a real value number in the range of 0–1, where zero indicates that the patient is unlikely to have cancer and 1 indicates that the patient is likely to have cancer.

### Effect of sample size on prediction accuracy

The limited sample size and large number of features meant removing patients from the dataset could negatively impact prediction accuracy. To evaluate the effect of sample size on accuracy, we randomly selected subsets of patients (without replacement) and trained a model on each subset. This process was repeated five times for each subset size. A linear model was used to analyse the relationship between the area under the curve (AUC) and sample size.

### Removal of patients who do not query for health-related topics

Not all internet users use Google for health information. It is hypothesized that prediction of health outcomes for these users will be more challenging. Therefore, we investigated the effect on prediction accuracy of removing users who did not query health-related online searches.

For a given time period commencing at T_1_ and ending at T_2_, patients were removed if they did not mention any of 5521 medical conditions [23] in the first half of the period, i.e. from T_1_ to (T_1_ + (T_2_-T_1_)/2). We only considered the first half, as we hypothesized that filtering on the second half might remove more benign patients and bias our results. A model was trained and evaluated as discussed previously. AUC is dependent on sample size (for small datasets) [35, 36]. To compensate for the decreased training set size, we normalise the resulting AUC according to the regression parameters described in supplementary Fig. 2 (following the approach described in Floares et al. [37]) We refer to this value as the sample-corrected AUC.

### Symptoms questionnaire as outcome predictors

The overlap between a patient querying about a symptom and mentioning it in the symptom questionnaire was evaluated by first mapping a list of query terms to each symptom. This is listed in the Supplementary information. Then, for each symptom, a 2 × 2 contingency table was calculated enumerating the number of patients that indicated (did not indicate) the symptom in the questionnaire and the number of patients that searched (did not search) for the symptom. The association was evaluated through the chi-squared test.

An indication of whether each patient mentioned a symptom was used as input to a prediction model of patient outcome, with and without the vector-space features. Thus, we compared the performance of a predictive model, using search queries only, then questionnaire symptom data only, and finally search queries and symptom questionnaire data combined. By comparing the performance of these three approaches, we can determine whether search activity trends are a superior risk indicator compared to questionnaires, as well as assess the potential added value of modelling both information sources together. The prediction model (gradient boosting) was used for all models.

Note that the questionnaire-based approach is less sensitive to sample size (owing to its low dimensionality): A regression model of AUC as a function of sample size was not statistically significant (*P* > 0.05). Therefore, the AUC of this model was not corrected for sample size.

### Comparison to a general population

To compare our results to a general population of UK-based internet users, we examined the queries of 1.8 million UK-based online users of the Microsoft Bing search engine. The Bing cohort consisted of users who searched for at least one keyword from the medical key word list (Supplementary Table 2) and made at least one search query each month between October 2021 and September 2022. Since this control group was anonymous, it included both males and females. Bing users who asked about gynaecological cancers ten or more times during the data period (October 2021-June 2022) or the three-month period immediately following (July–September 2022), were excluded from the analysis to safeguard against including people with a pre-existing or new gynaecological diagnosis [38]. The remaining Bing population was assumed not to have an active gynaecological cancer diagnosis. There are no known statistically significant differences in the demographics between Google and Bing users [39].

The control (Bing) group consisted of users who searched for health terms that could be relevant to gynaecological cancers but were unlikely to have a gynaecological cancer. A model with T_1_ = -270 and T_2_ = -1 (trained on data from all participants- benign and malignant cases) was applied to search queries made in the data period (9-month period, Oct 2021-June 2022) by users in the Bing control group. The end point of the data period (June 2022) in the Bing group represents the day before GP referral in the Gynaecology group.

## Ethical considerations

Institutional review board approval was granted in May 2020, by the North of Scotland ethics committee (REC approval 20/NS/0063). All patients signed informed consent to participate in the study. The filtered Google takeout files were pseudo-anonymised, and the original Google takeout file (non-filtered) was deleted. Data was processed in line with GDPR regulations. Permission was granted to utilise Bing data by the Microsoft Ethics Review Board (approval number 10532).

## Results

### Google use among gynaecology patients and acceptability of online search data use

77.3% (652/844) of individuals approached to participate in this study had a Google account. Of those who met the study inclusion criteria, 60.1% consented to study participation (392/652). Complete enrollment (Google Takeout file and completion of questionnaire) was achieved in 65.1% of individuals (255/392). Of those who completed enrollment (*n* = 255), 7.8% (20/255) were excluded due to insufficient online search data (i.e., no searches which passed a filter of health-related queries), resulting in a final dataset of 235 women (Fig. 1).

The 235 women in the cohort made 519,048 health-related queries (an average of 2208 searches per patient). The rate of malignancy was 26.0% (61/235), with predominantly ovarian (*n* = 42, 68.9%), followed by endometrial (*n* = 15, 24.6%) cancer. The cohort consisted of pre-dominantly post-menopausal women (*n* = 136, 57.9%) with a median age of 53 years old (20–81) (Table 1).

**Table 1:** Summary of demographics, clinical diagnoses and symptom presentation of individual’s referred with suspected gyncaecological cancer

**Total *N* = 235**	**Median, (range)**		
**Age**	53 (20–81)		
**BMI**	25.9 (16.5–50.0)		
		**Number**	**%**
**Ethnicity**	White European	141	60.0
	Asian	25	10.6
	Black/African	28	11.9
	Hispanic	6	2.6
	Mixed	9	3.8
	Indian	12	5.1
	Arab	8	3.4
	Other	6	2.6
**Parity**	0	88	37.5
	1	48	20.4
	2	58	24.7
	3	41	17.4
**Menopausal status**	Pre	99	42.1
	Post	136	57.9
**Diagnosis**	Benign	82	34.9
	Benign (histological)	92	39.1
	Malignant	61	26.0
**Malignancy type**	Ovarian	42	68.9
	Endometrial	15	24.6
	Cervical	1	1.6
	Gastrointestinal	3	4.9
**Symptom presentation**	Pelvic pain	150	63.8
	Bloating	119	50.6
	Post-menopausal bleeding	99	70.7
	Dyspareunia	62	26.4
	Weight loss	43	18.3
	Constipation	65	27.7
	Appetite loss	65	27.7
	Diarrhoea	44	18.7
	Urgency	63	26.8
	Frequency	91	38.7

The reasons for incomplete enrolment (*n* = 137, 34.9%) included: technical issues exporting the Google takeout file (27.6%, *n* = 108), study withdrawal (*n* = 12, 3.1%) and not tracking Google searches, so a takeout file (*n* = 17, 4.3%) could not be generated (Fig. 1). The excluded cohort (*n* = 137) had a median age of 51 years old (range: 20–89), which is comparable to the enrolled cohort. The excluded cohort had a rate of malignancy of 18.2% (*n* = 25), with 52% ovarian (*n* = 13), 40% endometrial (*n* = 10) and 8% non-gynaecology cancers (*n* = 2) respectively.

### Clinical symptoms present at different points in gynaecological Cancer

**Fig. 2:**
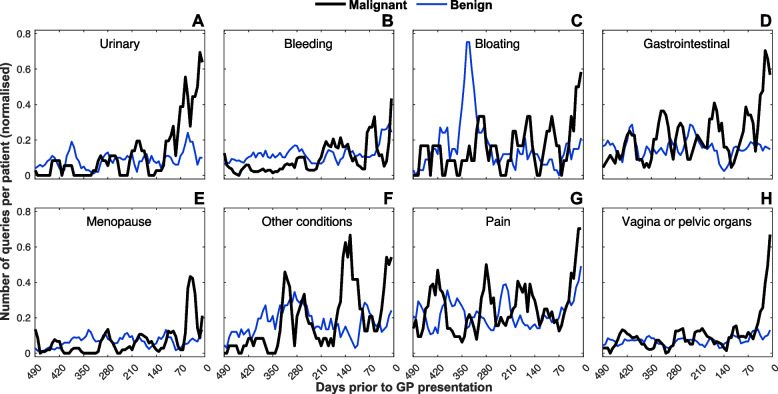
The time series chart outlining the number of online search queries per discrete category within the study cohort. The time series chart outlines the number of online search queries made per patient, within each distinct symptom category: menopause, urinary, bleeding, bloating, gastrointestinal, vagina, pain etc. stratified by outcome (benign/malignant) up to 490 days in advance of GP referral. The time series are smoothed using a 4-week moving average. Online search activity can identify symptomatic individuals with gynaecological cancer at an earlier stage

To evaluate the pattern of symptom presentation, the frequency of queries per week for each of the 14 categories in Supplementary Table 2 were evaluated and stratified by clinical outcome (malignant vs benign). Figure 2 shows the number of search queries within each category according to clinical outcomes. Gastrointestinal and pain-related symptoms presented up to 365 days before referral by the GP, whereas urinary and bleeding related symptoms presented later, at 140 days prior to GP referral. Around 70 days prior to GP referral, symptoms relating to bloating, gynaecological organs (vagina, pelvis) and menopause become more prevalent. The same pattern in symptom presentation was not seen within the benign group, thus suggesting a pattern that may be specific to malignancy.

**Fig. 3:**
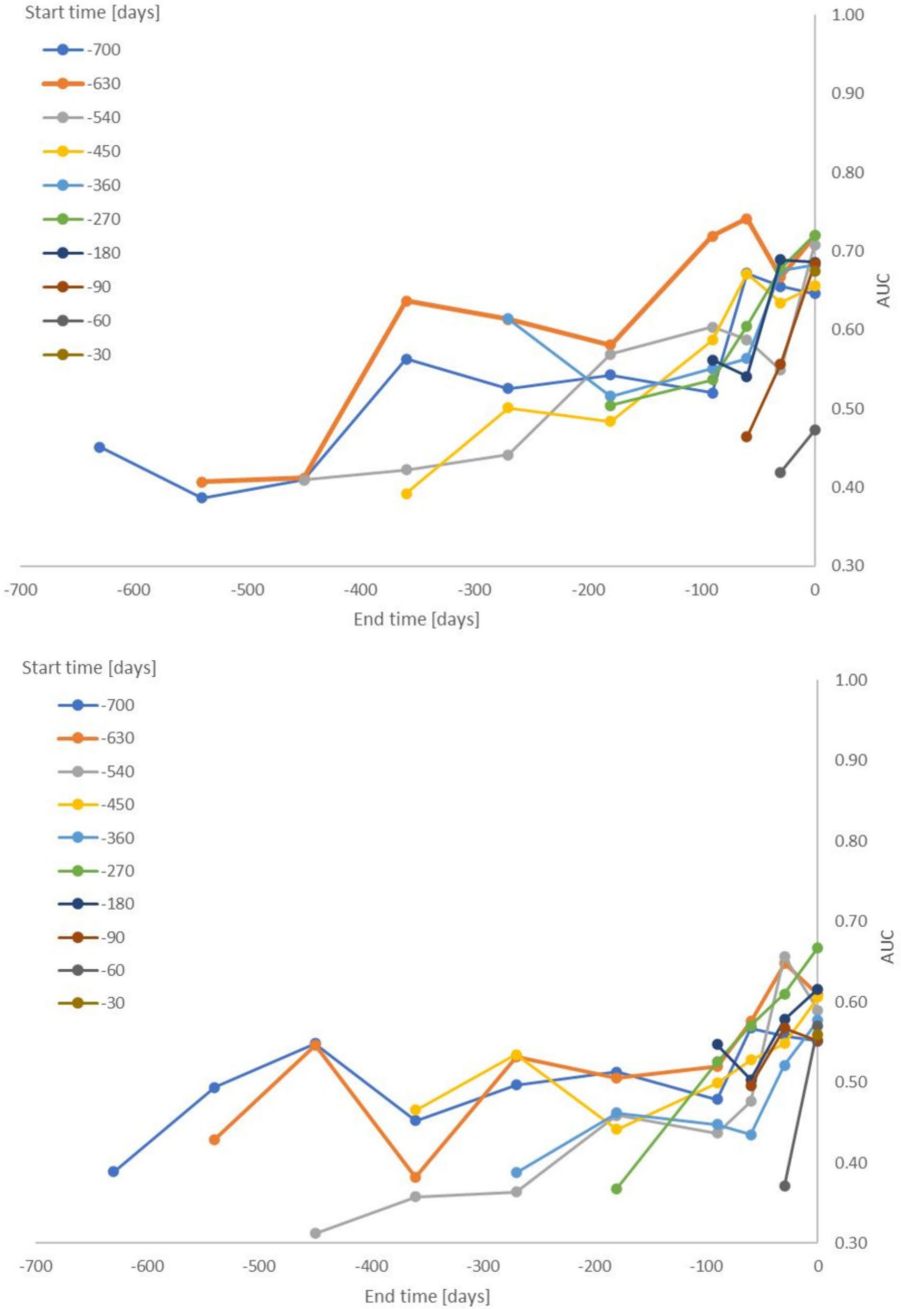
Model performance as a function of start and end times. The top figure shows the AUC for the terms model and the bottom figure for the categories model. The start and end times correspond to the duration of time in advance of GP referral date. Different lines correspond to different start (T_1_) times and the dots on each line correspond to different end (T_2_) times. Each dot represents the average of 10 runs. Standard deviation is equal, on average, to 0.01 (1.8% of the average AUC)

Queries (represented as either search terms or categories) were evaluated for various time windows. The start time (T_1_) was progressively increased from 30 to 700 days prior to the time of GP presentation. For each start time, the end time (T_2_) was progressively decreased i.e., length of the window was increased (Fig. 3). The model’s ability to differentiate between benign and malignant cases was evaluated based on the AUC. Across all time frames (T_1_, T_2_), the AUC for models using search terms representation was higher than models using categories representation by an average of 0.06 (*P* < 10^–5^, sign test) (0.11 when only including models with an AUC greater than 0.55, *P* < 10^–5^, sign test). Although both representations (search terms and categories) had comparable qualitative performance metrics (Fig. 3), we focused on the search terms representation given its superior quantitative performance.

Focusing on the ‘search terms’ model with a window starting 630 days before presentation to the GP, we observed the AUC surpass random decision (AUC 0.50) 360 days before the GP referral date (T_2_ = 360) (AUC 0.64). This suggests there may be a difference between health-related queries in benign and malignant cases as early as a year prior to GP presentation. The closer to the GP referral date, the better the model performed, as demonstrated by an AUC of 0.74 for a time window up to 60 days in advance of GP referral (T_1_ = 630, T_2_ = 60) (Fig. 3)*.*

The cohort contained some individuals (*n* = 82, 34.9%) who did not query any of 5521 pre-defined health conditions, i.e., they did not use online search for health purposes. These users most likely reduce the model’s performance. After removing 82 users who did not query any health condition, the AUC for the remaining 153 users (malignant *n* = 41, 26.8% and benign *n* = 112, 73.2%) for the time window up to 60 days in advance of GP presentation (T_1_ = 630 toT_2_ = 60) reached a sample-size adjusted AUC of 0.82. The sample size adjustment accounts for the fact that the AUC declines with sample size (for small samples) and is described in detail in the supplementary information.

The most common symptom in the patient survey was pain (*n* = 150, 63.8%), followed by bloating (*n* = 119, 50.6%) Table 1, which is in accordance with the NICE guidance for the typical clinical presentation of ovarian cancer [40]. In post-menopausal individuals (*n* = 136), the most common symptom was post-menopausal bleeding (*n* = 99, 70.7%).

Interestingly, no correlation was noted between online search and clinical symptom patterns when a chi squared test (*p* < 0.05 with Bonferroni correction) was applied. The lack of correlation between online search and clinical symptoms suggests that online search data is not a mere online record of their clinical symptoms. Instead, it appears to represent other important health data, that is not reflected in the healthcare questionnaire. The difference may also be attributable to the fact that questionnaire data, unlike online search data, relies upon an individual’s recall ability.

To assess the value of symptom questionnaire data in the detection of gynaecological malignancy, a model utilising: (1) questionnaire symptom data only and (2) questionnaire and online search queries (combined) was developed. The questionnaire-only model performance reached an AUC (sample corrected) of 0.62, when combined with online search terms the AUC improved to 0.77 (sample corrected, T_1_ = 630, T_2_ = 0). The addition of questionnaire data to the model appears to slightly decrease the performance of the online search query-based model from an AUC (sample corrected) of 0.82 to 0.77 respectively.

### Comparison to control (Bing) population

**Fig. 4:**
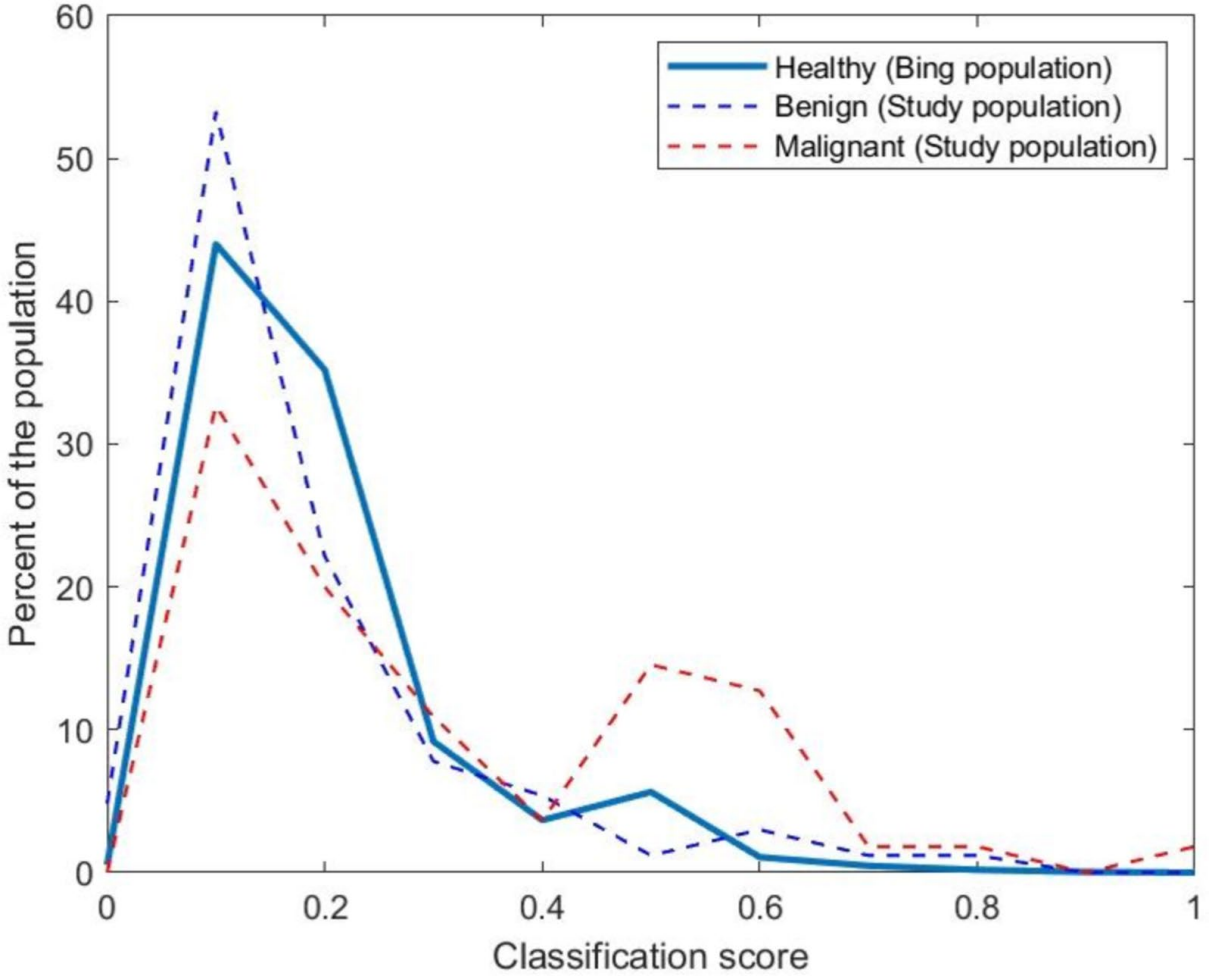
A histogram (10 bins) of model classification scores, when applied to our sample population (*n* = 235) and Bing users (*n* = 1.8 million). The histogram demonstrates the classification score for individual users. A high classification score indicates an increased likelihood of a malignant diagnosis. The Bing user population is distributed towards lower classification scores, in line with benign sample population and a lower likelihood of malignancy

The distribution of model scores for users in the control (1.8 million UK-based online users of the Microsoft Bing search engine), benign, and malignant groups are depicted in Fig. 4*.* The scores for the benign and malignant populations are computed using leave-one-out cross-validation, whereas a model trained on all benign and malignant patients was used for computing the model scores of the control population. The control group closely mirrors the benign study population (Fig. 4). This supports the potential generalisability of the model and its ability to discriminate individuals with benign and malignant diagnoses.

## Discussion

This is the first clinical pilot study to demonstrate the feasibility of using online search histories in individuals with known gynaecological diagnoses, as a potential disease detection tool. Our study suggests that screening based on online search data may provide a signal of disease, up to 360 days prior to primary care (GP) referral with a suspected malignancy (AUC 0.64) and gradually improves closer to the GP referral date. The best performing model had a sample size-adjusted AUC of 0.82 in users (*n* = 153) who engaged in health-related searches, up to 60 days prior to GP referral. Furthermore, online search data provided insight into the presentation of gynaecological cancer, with an increased frequency and severity of urinary and gastrointestinal symptoms noted around 140 days and menopausal symptoms and pain at around 70 days in advance of GP referral. The presence of symptoms up to a year in advance of a diagnosis of ovarian cancer is consistent with previous studies and challenges the concept that it is a ‘silent killer’ where most women are asymptomatic [41, 42]. This study is novel as it (1) evaluated online search data in individuals with known malignant and benign gynaecological conditions to ensure it is clinically robust and reproducible (2) used a symptomatic, benign cohort as a control group (3) and has a digital timeline of GP referral to diagnosis, to extract relevant time-specific data. Previous studies [25, 32–34] used proxy indicators such as experiential (or self-identifying) queries to identify individuals with the disease of interest, selected non-matched population controls and had no record of the diagnosis timeline.

We considered classification based on known health categories (categories model) and found that they were predictive. This is a validation of our data against medical knowledge, which is important. However, the model which used all search terms (terms model) outperformed the health categories model, which suggests that an individual’s ‘search terms’ are likely to encode additional variables, that may be important in disease detection and beyond our existing knowledge of disease. Furthermore, the lack of correlation between the questionnaire and the search term model reinforces the hypothesis that online search data is not a mere online representation of an individual’s symptoms, but incorporates other variables (i.e., nutrition, health behaviour) that are relevant in the risk of disease. The difference may in part be attributable to an individual’s ability to recall symptoms for up to 12 months in advance of disease presentation. Clinically, online search data may be a useful diagnostic adjunct in primary care to identify those at risk of disease and appropriately triage patients. Future work should focus on evaluating the performance of the search terms model in an independent population to understand its generalisability and potential clinical value as a diagnostic tool. Further validation will add to existing knowledge of clinical disease presentation and how it differs between benign and malignant conditions.

An individual’s online search data is an example of a digital footprint i.e., information people knowingly or unknowingly generate when using electronic services, including mobile telephone data, social media posts, credit and loyalty card use. The recent CLOCS study [43] evaluated loyalty card purchases within two retailers in 153 women with ovarian cancer, compared to healthy controls, to determine if shopping habits could be used as proxy symptoms indicators, to facilitate the earlier detection of disease. Whilst an association between ovarian cancer and purchases of over-the-counter indigestion (AUC 0.65) and pain (AUC 0.63) medications were identified up to 13 months before diagnosis, the results must be interpreted with an element of caution, given the control group consisted of healthy population rather than symptomatic women with a benign condition. Furthermore, online search data, likely hold more promise than loyalty card purchase data due to its relative accessibility, breadth of topic coverage and its frequency of use [43, 44].

Systematic delays in the referral pathway need to be addressed in order to facilitate earlier detection of gynaecological cancer [7]. This study provides a unique insight into the disease trajectory of gynaecological cancer and its typical presentation, which is invaluable for improving disease detection at a patient and primary care level, particularly given GP’s typically see an ovarian cancer case every five years [8, 45, 46]. Understanding the 'triggers’ for accessing primary care is useful from a health promotion perspective, given we know existing cancer campaigns do not generate sustained behavioural changes [47, 48]. Screening programs identify ‘at risk individuals’ and feed them directly into specialist services, thus circumnavigating referral delays [10]. The best performing online search-based model reached a sample-corrected AUC of 0.82, which is comparable to other established cancer screening programs in place to detect cervical (HPV, AUC: 0.87) and breast (Mammography AUC: 0.88) cancer [49, 50]. The test sensitivity is dependent on the operating point along the ROC. For example, the model with T_1_ = 0, T_2_ = 270 detects 36% of the positive cases at the cost of 8% false positives, while at another operating point, it can detect 62% of the positive cases at a cost of 38% false positives. The selection of the specific operating point is always a trade-off between the true positives and false positives and it depends on the specific clinical scenario where the model is used. For this reason, we focused on the AUC, which is an overall measure of performance, which takes all possible operating points into account.

This is the first study to evaluate online search data in individuals with known gynaecological conditions and linked symptom data. Substantial efforts were made to develop a generalisable model and reduce the risk of overfitting, through methodical leave one-out-cross validation and evaluation of the model’s performance in an independent control population. The next step should be to test the model in an independent test set of symptomatic women with linked gynaecological diagnoses to evaluate its clinical value as a diagnostic tool.

The date of GP referral into secondary care was used as the last date of online search data, given it was available and did not rely upon patient recall. However, the GP referral date may not reflect previous presentations to the GP (for the same condition), so could have introduced a degree of bias to the search patterns. Future work should collaborate with primary care to use the first date of presentation to the GP, to control for this bias. Furthermore, the use of multiple Google accounts or private browsing may have affected the quality of an individual’s online search data. Finally, the prevalence of malignancy within this cohort is not reflective of the UK population, given ovarian is less common (2%) than endometrial (2.78%)cancer [1, 3].

An online search-based model has potential as an accessible real-time screening tool, providing individualised risk profiles, which addresses barriers to screening uptake. We must acknowledge the physical and psychological morbidity and costs associated with a screening program triggering further investigations and treatment including surgery, particularly for those without the disease (false positive cases), when evaluating the value of an online search-based screening model within the health service [11, 49, 50]. There are several issues associated with using a model based on online search data. First, the digital divide may exacerbate health inequalities within disease screening programs [9]. Second, data anonymity and confidentiality are vital given the sensitive nature of online search data but could be addressed using cryptographic methods. Third, the psychological implications associated with receiving information suggesting a ‘high risk of cancer’. Further research into behavioural psychology is required to better understand how to manage these issues before clinical integration can be considered.

Finally, we have shown that online search data may be able to identify individuals with gynaecology cancer at an earlier point than standard care, which is comparable to the findings from previous ovarian cancer screening trials (UKTOCs and PLCO) [12, 51]. Whether earlier detection of disease translates to improved clinical outcomes, i.e., mortality benefit, needs to be evaluated in a sufficiently powered clinical study, with adequate malignant cases to understand its clinical value as a diagnostic support tool. Furthermore, validation studies will contribute to existing knowledge about clinical disease presentation, thus supporting the discrimination between malignant and benign conditions.

## Conclusion

This is the first study to demonstrate the potential role of online search data in facilitating the earlier detection of clinically confirmed disease, specifically, though not limited to gynaecological cancer. Predictive performance varied depending on whether categorical or uncategorized ‘search terms’ were used. The best search-terms based model had a comparable performance to established disease screening programs [49, 50]. However, further research is required to evaluate the performance of the online search model within a larger cohort. Our results demonstrate the feasibility and acceptability of utilising online search data for health screening, which highlights its potential application in other diseases. To further evaluate the diagnostic capability of online search data in the earlier detection of disease, we aim to do a multi-centre study, to improve the overall performance and generalisability of the model to the general population, thus supporting its translation into clinical practice.

### Supplementary Information

**Additional file 1:** **Supplementary Figure 1. **Clinical questionnaire (Page 1-3) to extract clinical data, including symptom presentation, medical, family history and social history. **Supplementary Table 1**. Outlines the specific questionnaire symptoms that link to defined keywords identified in the online search query data. **Supplementary Table 2.** Outlines the list of online search query keyword categories, the number of queries containing the specific keywords and the three most common queries in each category in English, Spanish, and French. **Supplementary Figure 2.** Outlines the dependence of model AUC on sample size. The dotted line is a linear regression curve whose parameters are shown in the figure. This regression curve was used to assess the sample-size-adjusted AUC.

## Data Availability

The datasets generated and/or analysed during the current study are not publicly available as patient permission for open access was not obtained but are available from the corresponding author on reasonable request.

## Abbreviations

OC: Ovarian cancer
EC: Endometrial cancer
OSD: Online search data
GP: General practitioner /primary care physician
AUC: Area under the ROC curve
UK: United Kingdom
UKCTOCS: UK collaborative trial of ovarian cancer screening
CA-125: Cancer antigen-125
NICE: National institute for clinical excellence
ROCkeTS: Refining ovarian cancer test accuracy scores
GT: Google takeout
NHS: National health service
BMI: Body mass index
REC: Regional ethics committee
GDPR: General data protection regulation
T1: Start time
T2: End time
CLOCS study: Cancer loyalty card study
