# Correction: Using online search activity for earlier detection of gynaecological malignancy

**DOI:** 10.1186/s12889-024-18831-0

**Published:** 2024-05-21

**Authors:** Jennifer F. Barcroft, Elad Yom-Tov, Vasileios Lampos, Laura Burney Ellis, David Guzman, Víctor Ponce-López, Tom Bourne, Ingemar J. Cox, Srdjan Saso

**Affiliations:** 1 Imperial College London, Hammersmith Hospital Campus, Du Cane Road, London, W12 0HS UK; 2 Microsoft Research, Hoshaya, Israel; 3 Department of Computer Science, University College London, London, UK; 4 Computer Science, University of Copenhagen, Copenhagen, Denmark

**Correction: BMC Public Health (2024) 24:608**

10.1186/s12889-024-17673-0

The original publication of this article contained an incorrect author name. The incorrect and correct information is listed in this correction article, the original article has been updated.

Incorrect

Vasilieos Lampos

Correct

Vasileios Lampos

