# Supplementary material for: Using online search activity for earlier detection of gynaecological malignancy
Source: BMC Public Health. 2024 Mar 11;24:608. doi: 10.1186/s12889-024-17673-0 (PMC10926628; doi:10.1186/s12889-024-17673-0)
Supplement: Supplementary file 1 — Additional file 1: Supplementary Figure 1. Clinical questionnaire (Page 1-3) to extract clinical data, including symptom presentation, medical, family history and social history. Supplementary Table 1. Outlines the specific questionnaire symptoms that link to defined keywords identified in the online search query data. Supplementary Table 2. Outlines the list of online search query keyword categories, the number of queries containing the specific keywords and the three most common queries in each category in English, Spanish, and French. Supplementary Figure 2. Outlines the dependence of model AUC on sample size. The dotted line is a linear regression curve whose parameters are shown in the figure. This regression curve was used to assess the sample-size-adjusted AUC. [file 12889_2024_17673_MOESM1_ESM.docx]

## **Supplementary Information**

**Supplementary Figure 1:** Clinical questionnaire (Page 1-3) to extract clinical data, including symptom presentation, medical, family history and social history.

**Clinical Questionnaire (page 1)**

***Demographics and Medical history***

Age | Height | Weight | Ethnicity

**Do you have any of the following conditions?** *If yes, please circle:*

Diabetes mellitus Low or High thyroid hormone (hyper/hypothyroidism)

Asthma Autoimmune disorders High blood pressure (hypertension)

**Have you been diagnosed with *covid-19 infection*?** (yes/no) *If yes, specify the date:*

***Gynaecological history***

**When did your periods first start (Menarche)?**

**Are you known to have any of the following *conditions*, if so *when* were you diagnosed**?

Endometriosis | Uterine Fibroids | Polycystic Ovarian Syndrome | Adenomyosis

**Have you had any previous *pelvic surgery*?** (*please circle all that apply*)

Uterine polyps | Fibroids | Ovarian Cysts | Endometriosis | Tubal

Uterine Septum | Hysterectomy (removal womb) | Oophorectomy (remove ovary)

**Do you have a history of *pelvic infection (i.e., Chlamydia/Gonorrhoea)*?** (yes/no)

**Have you undergone any gynaecological investigations?** (yes/no)

Pelvic ultrasound | Endocrine screen | Tumour markers | Diagnostic laparoscopy

***Pre-Menopausal:***

**Do you currently use any contraception or have done in the last year?**

Combined pill | Progestogen only pill | Natural cycle | Mirena Coil

Nexplanon | Copper IUD | Condoms

**Do you have regular periods?** (yes/no)

**How long is your usual menstrual cycle (cycle start = first day of period)?**

**How many days does your period normally last for?**

**Have your periods currently stopped for more than 6 months (Amenorrhoea)?** (yes/no)

**Do you have to take time off work or use regular pain killers due to your period?** (yes/no)

**Have you experienced any of the *following symptoms*?** (*please circle all that apply*)

Excess hair (face, arms) | Acne | Loss of hair (head)

***Fertility***

**Are you currently wishing to conceive?** (yes/no)

**Do you have any history of subfertility?** (yes/no), *if so how long have you been trying to conceive?*

**Have you undergone fertility treatment previously?** (yes/no)

**Have you undergone treatment for *recurrent miscarriage*?** (please circle all that apply)

Progesterone | Aspirin | Steroids | Low Molecular Weight Heparin

**Have you undergone any *complementary therapies* (acupuncture, reflexology)?**

**Has your partner undergone *semen analysis?*** (yes/no)

*if yes, was the result: Normal| Abnormal (specify abnormality: ……………….)*

**Clinical Questionnaire (page 2)**

***Pregnancy History***

**Are you currently pregnant?** (yes/no)

**Have you ever been pregnant?** (yes/no). *If yes outline in table below*:

| **Date** | **Pregnancy outcome (miscarriage, ectopic, live birth, ongoing pregnancy, termination)** | **Mode of delivery (caesarean, vaginal)** |
| --- | --- | --- |
|  |  |  |
|  |  |  |
|  |  |  |
|  |  |  |

***Post-menopausal:***

**What age did you go through the menopause?**

**Have you ever used hormonal replacement therapy?** (yes/no)

*If yes which regimen, are you currently using or most recently (if stopped):*

Cyclical (bleed regimen) | Continuous regimen (no bleed regimen)

***Gynaecological Cancer***

**Are you up to date with your cervical smears?** (yes/no)

**Have you ever had any treatment on your cervix?** (yes/no)

**Do you have a personal history of *female-specific cancer*?**

Breast Cancer | Ovarian cancer | Uterine (womb) cancer | Cervical cancer

**Have you ever used tamoxifen?** (yes/no)

***Social History***

**Do you smoke?** (yes/no/ex-smoker)

**Do you drink alcohol?** (yes/no)

**Do you ever use recreational drugs?** (yes/no)

**Do you regularly drink caffeinated drinks**? (yes/no)

**How often per week do you exercise?**

***Family history*:**

Do any first-degree relatives (mother, sister, child) have ovarian cancer?

Do any first-degree relatives (mother, sister, child) have endometrial cancer?

***Mental Health***

Have you felt your *mental health* has been affected by your gynaecological symptoms? If yes, please circle all that apply: Low mood | Anxiety | Stress

**Clinical Questionnaire (page 3)**

| Have you experienced the following symptoms in the last 12 months? | Y/N | When did the symptoms start? | How often do you experience the symptoms?  (daily, 1-5/week, fortnightly, monthly) | At their worst, how severe are your symptoms?  (1-10 with 10 being the most severe |
| --- | --- | --- | --- | --- |
| Pelvic pain |  |  |  |  |
| Bleeding after sexual intercourse |  |  |  |  |
| Increased abdominal size (girth) |  |  |  |  |
| Abdominal bloating |  |  |  |  |
| Appetite loss |  |  |  |  |
| Constipation |  |  |  |  |
| Diarrhoea |  |  |  |  |
| Sudden surge to pass urine (urgency) |  |  |  |  |
| Passing urine more frequently (frequency) |  |  |  |  |
| Passing urine at night (nocturia) |  |  |  |  |
| Weight loss |  |  |  |  |
| Weight gain |  |  |  |  |
| Feeling full (early satiety) |  |  |  |  |
| Reflux |  |  |  |  |
| Pain during sex (Dyspareunia) |  |  |  |  |
| Change in vaginal discharge |  |  |  |  |
| *If pre-menopausal, do you experience:* |  |  |  |  |
| Heavy periods |  |  |  |  |
| Bleeding in between periods |  |  |  |  |
| Painful periods (dysmenorrhea) |  |  |  |  |
| Pain when opening bowels (dyschezia) |  |  |  |  |
| *If post-menopausal, do you experience:* |  |  |  |  |
| Bleeding after the menopause |  |  |  |  |

| Symptom | Keywords |
| --- | --- |
| Appetite loss | appetite loss, lost appetite, no appetite, food aversion, loss of appetite, no appetite |
| Constipation | passing stool, bowel, constipation, constipated, obstipation, obstipate, blocked up, obstructed |
| Diarrhea | diarrhoea, diarrhea, loose bowel, loose motion, the runs, looseness for the bowels, the trots, flux, dysentery |
| Distension | bloat, girth, swollen, bulge, enlargement, distension, distention |
| Dyspareunia | dyspareunia, dyspareunia, pain during, painful intercourse, painful sex, discomfort during sex, vaginismus |
| Frequency | going to the loo, passing urine often, peeing often, pollakiuria, peeing frequently, going to the bathroom, frequent urin, urination per day |
| Pelvic pain | stomach, pelvic, pelvis, abdomen, abdomin, tummy, discomfort, throbbing |
| Urgency | urgency to pass urine, urgent urine |
| Weight loss | weight loss, lose weight, reduced weight, loose clothes, weight fall off |

**Supplementary Table 1.** **Outlines the specific questionnaire symptoms that link to defined keywords** identified in the online search query data.

| **Category** | **Number of queries** | **Keywords** | **Most common queries** |
| --- | --- | --- | --- |
| **Bleeding** | 1984 | bleed, blood, stain, sangr, mancha, tache, saigner, du sang | bleeding after menopause, crp blood test, new blood art |
| **Bloating** | 209 | bloat, swollen, gonfler, ballonnement, inflar, hinchazon, hinchar, gonfler, | bloated stomach, stomach hard and bloated, home remedies for bloating gas in stomach |
| **Diagnostics** | 91 | ultrasound, ca 125, ca125, ultrasonido, ultrason | abdominal and pelvic ultrasound, ca 125 what is considered high, transvaginal ultrasound |
| **Fatigue** | 770 | fatigue, tired, exhaust, malaise, sleep, fatiga, cansad, epuis, malestar, dormir | before I go to sleep, sleep sounds, sleep disorder clinic |
| **Gastrointestinal** | 724 | blood in stool, passing stool, bowel, constipation, reflux, diarrhoea, vomit, feeling sick, satiety, appetit, hunger, ibs, irritable, intestin, intestino, estrenimiento, reflujo, diarrhee, diarrea, diarrhea, vomir, votacion, sentirse enfermo, se sentir malade, satiete, saciedad, apetito, hambr, faim | bowel cancer symptoms, bowel cancer, inflammatory bowel disease |
| **Gynaecological conditions** | 330 | fibroids, endometrios, pcos, kyste, ascites, cyst, prolaps | endometriosis, fibroids, ascites |
| **Menopause** | 575 | perimenopaus, menopause, postmenopausal | menopause symptoms, perimenopause, menopause |
| **Nutrition** | 4562 | recip, tesco, sainsbury, asda, morrison | morrisons, morrison, tesco near me |
|  | 1444 | domino, mcdonald, kfc, pizza hut, restaurant, subway, pizza express, sonic, starbucks, open table, uber eats | uber eats, kfc order online, restaurants |
| **Other conditions** | 461 | bowel cancer, deficiency, diverticulitis, descarga, decharge, mycose, cancer intestinal, cancer de l'intestin, carence, deficiencia, diverticulite, quiste, stones | bowel cancer symptoms, diverticulitis, bowel cancer |
| **Pain** | 2465 | pain, ache, stabbing, douleur, dolor, poignarder, punalada | ovulation pain, lower back pain, abdominal pain |
| **Pelvic organs** | 369 | ovary, ovari, endometrial, womb, peritoneal, , ovaire, ovario, uterus | ovarian cancer symptoms, symptomes cancer ovaires, ovarian cancer |
| **Symptoms** | 2926 | symptom, diagnosis, sign, sintoma, diagnostic, diagnostico | ovarian cancer symptoms, bowel cancer symptoms, menopause symptoms |
| **Urinary** | 324 | bladder, urin, dysuria, vessie, dysurie, disuria, vejiga, orina calculos, calculs | urinary tract infection, bladder cancer, blood in urine |
| **Vagina or pelvic organs** | 999 | vagin, stomach, pelvic, pelvis, abdomen, abdomin, tummy, estomac, estomago, pelvien, bassin, barriguita, ventre discharge, yeast infection candidiasis | stomach cancer symptoms, bacterial vaginosis, bloated stomach |

**Supplementary Table 2.** **Outlines the list of online search query keyword categories**, the number of queries containing the specific keywords and the three most common queries in each category in English, Spanish, and French

**Supplementary Figure 2.** **Outlines the dependence of model AUC on sample size**. The dotted line is a linear regression curve whose parameters are shown in the figure. This regression curve was used to assess the sample-size-adjusted AUC.


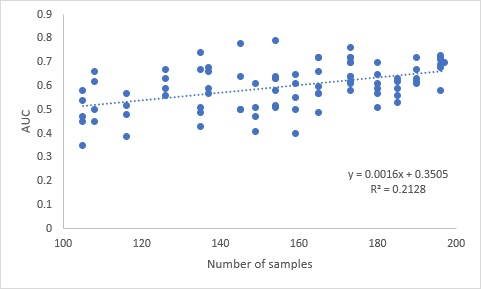


### **Analysis of terms with the highest weight**

We manually analysed the most indicative attributes of the search terms model. Specifically, we analysed the model applied to the Bing data (see Methods) by calculating the 50 terms with the greatest feature importance from the model. See the documentation for Gradient Boosting Classifier in sklearn version 1.0.2 for the computation of feature importance.

It is important to note that, since the Gradient Boosting Classifier is a non-linear model, feature importance is a measure of the effect of each attribute on the classification, not on whether that effect is positive or negative. Moreover, since the model allows for interactions among attributes, the effect can be dependent on the appearance of other attributes (or lack thereof).

We extracted all queries (regardless of patient outcome) that contained one or more of the 50 terms. We then ranked the queries based on their frequency of occurrence and one of the authors examined the 20 most common queries containing each of the 50 terms. A term was marked as related to gynecological cancers if one or more of the most common queries was clearly related to the gynecological cancers (e.g., was a relevant symptom, referred to a medical facility or a relevant medical test, etc.).

Of the 50 search terms with the greatest importance, only 20 (40%) were related to gynecological cancers. This highlights the complexity of disease prediction models and the potential value from extending beyond the ‘traditional’ symptom, based approach to identifying those at risk of a specific disease.
